# Recovery Time Following Continuous Midazolam Infusion During Dental Implant Surgery: A Prospective Observational Study

**DOI:** 10.7759/cureus.101819

**Published:** 2026-01-19

**Authors:** Naoko Murata, Yoshiki Shionoya, Shin Ogura, Katsuhisa Sunada

**Affiliations:** 1 Department of Dental Anesthesiology, The Nippon Dental University School of Life Dentistry, Tokyo, JPN; 2 Department of Oral Implant, The Nippon Dental University Hospital, Tokyo, JPN

**Keywords:** ambulatory anesthesia, ambulatory surgical procedure, anesthesia recovery period, conscious sedation, continuous intravenous infusion, dental implants, midazolam

## Abstract

Introduction: This study measured recovery time following sedation with a continuous midazolam infusion during implant surgery.

Methods: In this prospective observational study, we evaluated American Society of Anesthesiologists (ASA) I or II patients who underwent sedation with continuous midazolam infusion during implant surgery. The recovery time was measured at Nippon Dental University Hospital from December 2019 to July 2021. Patients under 18 years old with a history of drug allergies, obesity (body mass index of 26 kg/m^2^ or more), or regular use of antipsychotic, psychotropic, or hypnotic substances were excluded from the study. Midazolam was administered as a bolus dose of 0.05 mg/kg, followed by a continuous infusion to maintain a bispectral index (BIS) of 70-80. Midazolam was discontinued at the end of the procedure, and the awakening time was defined as that when the BIS was ≥90. After transfer to the recovery room, the patient’s condition was assessed using the postanesthetic discharge scoring system (PADSS) and then reassessed every 20 minutes. When the score reached ≥9, the patient was allowed to return home. The elapsed time from initiation of midazolam to when the PADSS score exceeded 9 was 90-110 minutes. Covariate variables were collected, including age (years), gender (male or female), weight (kg), local anesthetics (mL), surgical and sedation time (minutes), and induction and total dose of midazolam (mg).

Results: Forty-six patients participated in the study. Patients had a mean age of 59.4 (±12.1) years, a male-to-female ratio of 20:26, and a mean weight of 59.5 (±11.6) kg. Patients could be discharged within approximately 90-110 minutes of the start of midazolam administration.

Conclusion: If the 50 minutes of conscious sedation with midazolam were properly controlled, patients could be discharged within approximately 90-110 minutes of the start of midazolam administration. This means they can return home within 40-60 minutes after awakening.

## Introduction

Dental implants are common treatment options in modern dentistry. However, they are associated with anxiety and psychological stress. Local anesthesia alone is sometimes insufficient for dental implant surgery; therefore, intravenous sedation may be selected [1]. Propofol and other benzodiazepines, especially midazolam, are commonly used sedatives because they are easily controlled and can decrease autonomic activation and hemodynamic changes associated with anxiety and surgical stress [2]. Propofol is one of the most frequently used intravenous sedative agents during dental procedures. Continuous infusion allows titration to the desired level of sedation and provides rapid recovery because of its short context-sensitive half-time and short effect-site equilibration time [3-5]. In dentistry, propofol is often administered for sedation during implant surgery [6-8]. However, because the anesthetic contains egg yolk lecithin and soybean oil [4], the use of propofol in patients who experience injection pain or are allergic to these substances raises concerns [5]. Midazolam is a widely used benzodiazepine that produces reliable hypnotic, amnesic, and anxiolytic effects owing to its pharmacological actions, including sedation, hypnosis, and anxiolysis. Its short duration of action compared with other benzodiazepines is also notable. Midazolam has a less stimulating effect on the vasculature and is more comfortable for patients than propofol [9-12]. For intravenous sedation, midazolam has previously been administered intermittently while the patient's condition is monitored. However, there is no clear indicator of the timing of additional administration; therefore, anesthesiologists typically rely on their personal preferences or a rule of thumb. Consequently, maintaining a constant depth of anesthesia is challenging and may lead to insufficient sedation or respiratory depression due to deep sedation. On the other hand, the short-acting nature of midazolam enables continuous administration, but it is not commonly used because clinicians may be concerned about the possible prolonged duration of its effects. Few studies have investigated the continuous administration of midazolam. Thus, the recovery time after continuous midazolam infusion following bolus administration during dental implant surgery was investigated in this study.

This article was previously presented as a meeting abstract at the 2025 International Association of Dental Research Annual Meeting on June 26, 2025 [13].

## Materials and methods

A prospective observational study was conducted at Nippon Dental University Hospital and the Division of Oral Implants in patients who underwent implant surgery under sedation from December 2019 to July 2021. All patients were classified as American Association of Anesthesiologists physical status I or II. Patients under 18 years old with a history of drug allergies, obesity (body mass index of 26 kg/m^2^ or more), or regular use of antipsychotic, psychotropic, or hypnotic substances were excluded from the study. This study was approved by the Ethics Committee of Nippon Dental University School of Life Dentistry, Tokyo, Japan (approval number NDU-T2016-14). The study was performed in accordance with the guidelines of the institution and the Declaration of Helsinki, and was conducted after clinical trial registration, as certified by the International Committee of Medical Journal Editors (trial identification number: UMIN 000025556). Informed consent was obtained from all participants included in the study.

Procedure, sedation, and monitoring

Patients presented to the dental hospital on the day of surgery, having appropriately fasted for eight hours and not consumed any clear fluids for two hours prior to arrival. No premedication was administered to any of the patients. Upon arrival in the operating room, anesthesia monitors consisting of noninvasive blood pressure, electrocardiography, pulse oximetry, and bispectral index (BIS) monitors (A-1050, Nihon Koden Corp., Tokyo, Japan) were applied. The BIS score prior to sedation was 90 or higher in all patients. Systolic blood pressure (SBP), diastolic blood pressure (DBP), heart rate, and percutaneous oxygen saturation (SpO_2_) were measured every five minutes. After administering oxygen at 3 L/minute via nasal cannula, intravenous access was secured using a 22-gauge catheter. Saline was added to midazolam (Dormicum injection 10 mg; Maruishi Pharmaceutical, Osaka, Japan) to prepare a 10 mL solution, which was administered via an infusion pump (TE-332S, Terumo Corp., Tokyo, Japan). A bolus dose of 0.05 mg/kg midazolam was administered as the initial dose, and the infusion rate was 1,200 mL/minute. This time point was defined as the initiation of anesthesia. If a BIS value between 70 and 80 was not achieved after the initial dose, additional doses were administered until a value between 70 and 80 was reached. Then, the continuous dose was initiated at 0.025 mg/kg/hour and was thereafter adjusted infusion dose to maintain a BIS of 70-80. Three minutes after the BIS value of 70-80 was achieved, local anesthesia (2% lidocaine containing 1:80,000 epinephrine) was administered, and an anesthetic solution was added when patients complained of pain during surgery. During the operation, continuous infusion of midazolam was made to maintain BIS 70-80 until the end of the procedure. Paracetamol (acelio Bag of Intravenous Injection 1,000 mg, TERUMO, Tokyo, Japan) was administered when the surgery was completed. Midazolam was discontinued at the end of the procedure, and the awakening time was defined as that when the BIS was ≥90. After transfer to the recovery room, the patient’s condition was initially assessed using the postanesthetic discharge score (PADSS) and subsequently reassessed every 20 minutes (Table 1) [14].

**Table 1:** Postanesthetic discharge scoring system BP: blood pressure; HR: heart rate

Parameter	Score
Vital signs	
BP and HR ± 20% of preoperative value	2
BP and HR ± 20%-40% of preoperative value	1
BP and HR ± 40% of preoperative value	0
Activity, mental status	
Steady gait, no dizziness or meets preoperative level	2
Requires assistance	1
Unable to ambulate	0
Pain, nausea, and vomiting	
Minimal	2
Moderate	1
Severe	0
Surgical bleeding	
None or minimal (not requiring intervention)	2
Moderate (1 episode of hematemesis or rectal bleeding)	1
Severe (≧2 episodes of hematemesis or rectal bleeding)	0
Intake and output	
PO fluids and voided	2
PO fluids or voided	1
Neither	0

When the score was ≥9, the patient was discharged home. Surgical time (time from the initiation of surgery to completion), sedation time (time from the initiation of sedation to awakening), awakening time (time from surgery to awakening), and midazolam dose were recorded. The primary endpoint was time to PADSS ≥9, and the secondary endpoint was the number of patients with oxygen saturation ≤90%. As this is a single-arm study, no null hypothesis was set.

## Results

Forty-six patients participated in the study (Table 2).

**Table 2:** Patient character and sedation data SD: standard deviation

Subject	Mean ± SD
Age (years old)	59.4 ± 12.1
Gender (M/F)	20/26
Weight (kg)	59.5 ± 11.6
Local anesthetics (mL)	5.1 ± 1.6
Surgical time (minutes)	31.2 ± 17.7
Sedation time (minutes)	51.3 ± 17.3
Awakening time (minutes)	11.3 ± 9.7
Induction dose of midazolam per weight (mg/kg)	0.05 ± 0.015
Total dose of midazolam per weight per hour (mg/kg/hour)	0.11 ± 0.06

The most common complication was hypertension, followed by hyperlipidemia and diabetes mellitus, all of which were well controlled. Blood pressure was controlled either by angiotensin II receptor blockers, angiotensin-converting enzyme inhibitors, or calcium channel blockers, maintaining levels below 140/90 mmHg. Hyperlipidemia was controlled by 3-Hydroxy-3-methylglutaryl coenzyme-A reductase inhibitors; therefore, low-density lipoprotein cholesterol was less than 140 mg/dL, high-density lipoprotein cholesterol was 40 mg/dL or more, and triglyceride was less than 150 mg/dL. Diabetes was controlled with sulfonylurea drugs, biguanides, dipeptidyl peptidase 4 inhibitors, α-glucosidase inhibitors, sodium-glucose cotransporter 2 inhibitors, and HbA1c <7.0%. One patient experienced an intraoperative SpO_2_ drop below 95%, which was promptly resolved by airway clearance with mandibular elevation; no patient dropped below 90%. The SBP did not fall below 80 mmHg in any patient. Thirty-nine patients had no intraoperative memories, and seven had fragmentary memories. The mean PADSS score exceeded 9 between 40 and 60 minutes after arrival in the recovery room, indicating that the elapsed time from initiation of midazolam to the PADSS score exceeding 9 was 90-110 minutes. The times to recovery of oral intake and urination were the longest (Table 3).

**Table 3:** Results of PADSS (mean ± SD) PADSS: postanesthetic discharge scoring system; SD: standard deviation

Time (minutes)	20	40	60	80	120
Vital signs	1.91 ± 0.35	1.96 ± 0.20	1.98 ± 1.14	2.00 ± 0.00	2.00 ± 0.00
Awareness and gait	1.28 ± 0.50	1.65 ± 0.56	1.96 ± 0.20	1.98 ± 0.15	2.00 ± 0.00
Pain and nausea and vomiting	1.98 ± 1.14	1.98 ± 1.14	1.98 ± 1.14	1.98 ± 1.14	1.98 ± 1.14
Bleeding	1.98 ± 1.14	2.00 ± 0.00	2.00 ± 0.00	2.00 ± 0.00	2.00 ± 0.00
Oral intake and urination	0.26 ± 0.48	0.87 ± 0.71	1.71 ± 0.57	1.94 ± 0.32	2.00 ± 0.00
Total points	7.38 ± 0.99	8.49 ± 1.20	9.66 ± 0.70	9.89 ± 0.48	9.98 ± 0.15

## Discussion

The results of the present study showed that when midazolam was administered continuously to maintain a BIS value of 70-80, and the patient was sedated for 50 minutes, the recovery state, as assessed by PADSS, showed an elapsed time from awakening to the ability to return home of 40-60 minutes. PADSS was reported by Chung; it evaluates a patient's ability to be discharged from the hospital after anesthesia based on vital signs, mental state, ambulation ability, bleeding condition, and water intake ability [14]. This finding indicates that the elapsed time from initiation of anesthetic administration to discharge home ranged from 90 to 110 minutes.

The blood concentration of midazolam at the time the patient returned home was calculated using AnestAssist (version 1.9; Palma Healthcare Systems LLC, WI). The patients had an average weight of 60 kg and an average age of 59 years. The total midazolam dose in the current study was 0.11 mg/kg/hour; the duration of administration was 51 minutes. Assuming all patients were men with an average height of 170 cm, the midazolam blood concentration at 40 minutes after awakening was 26 ng/mL, and at 60 minutes was 21 ng/mL. Considering that patients awaken at midazolam blood concentrations below 50 ng/mL, these results are reasonable. Luyk et al. [15] administered midazolam to 41 patients with a mean age of 22 years and a mean weight of 73 kg for 30 minutes of continuous infusion following bolus administration, and evaluated the recovery time by choice reaction time (CRT; this test could measure the quality of recovery from the midazolam sedation at the end of the procedure). The results of that study showed that the CRT was not significantly different from the pre-dose value in 90 minutes after administration (60 minutes after the end of administration). Total midazolam doses in the Luyk et al. study averaged 7.6 mg (95% CI: 7.0-8.3 mg). Assuming that all patients were men and had an average height of 170 cm, the midazolam blood concentration 60 minutes after treatment completion was 28 ng/mL, and at 90 minutes was 22 ng/mL. On the other hand, the critical flicker fusion threshold was evaluated, and it was not significant between the presedation value and 120 minutes after administration (90 minutes after the end of administration). Critical flicker fusion threshold is a measure of the ability to perceive changes in light. However, the CRT, which evaluates the reaction time to a stimulus, is more appropriate as an index of permission to return home. Thus, the results of our study are consistent with those of Luyk et al. In addition, a retrospective study by Van den Berg and Preckel [16] on continuous midazolam administration via a target-controlled infusion pump, with BIS as the index of sedation, during periodontal surgery and implant procedures showed that all patients returned home within 60 minutes after the end of administration, supporting the present results. Regarding the relationship between bolus administration and time to return home, McGimpsey et al. [17] administered midazolam at 0.1 mg/kg to 22 women with an average weight of 56 kg and reported an elapsed time of 63.2 ± 6.1 minutes to return to predose values as assessed by the pegboard test. In the study by McGimpsey et al., the blood midazolam level at recovery was 33 ng/mL, assuming an average patient height of 160 cm. Gupta et al. [18] also reported that midazolam at 0.1 mg/kg was administered to 11 healthy volunteers with an average age of 29 years, an average weight of 73 kg, and an average height of 180 cm, who were evaluated using computerized dynamic posturography and recovered to their predose values after 105 minutes. Assuming that all subjects in the study by Gupta et al. were men, the blood level at recovery was calculated as 22 ng/mL. Thomson et al. [19] also reported that after administering a bolus midazolam dose of up to 0.75 mg/kg to 20 patients with an average age of 26.8 years who were scheduled for extraction of an impacted third molar, all went home 60 minutes after surgery. On the other hand, Takarada et al. [20] reported that 10 patients with a mean weight of 51 kg, mean height of 162 cm, and mean age of 46 years received a midazolam bolus of 0.063 ± 0.036 mg/kg for an average of 33 minutes of dental treatment, and the median time from the end of treatment to permission to return home was 55 minutes (range: 20-175). In the study by Takarada et al., assuming that all patients were men, the midazolam blood level at the time of returning to home was 15 ng/mL. However, comparing these results with those of the present study is challenging because objective criteria for permission to return home were not provided. These findings indicate that when patients are sedated by midazolam continuous infusion for approximately one hour, continuous infusion does not prolong the time before they are allowed to return home because blood levels are similar in the one hour after awakening to the standard bolus administration protocol, as long as the appropriate level of sedation is maintained. The relatively short context-sensitive half-life of midazolam is thought to be the reason for its rapid decrease in blood concentration after short, continuous dosing.

In a previous study evaluating recovery time during intermittent administration, Pratila et al. [21] reported that 29 patients, with an average age of 47 years and an average weight of 70 kg, received intermittent midazolam administration. The Pratila et al. study reported a sedation time of 50 minutes and a total midazolam dose of 4.79 mg, similar to the present study, but patients were able to stand unassisted in 90.8 ± 29.9 minutes after the end of administration. The blood level at this point was approximately 10 ng/mL, which is not consistent with our results. Fujisawa et al. [22] also reported that after intermittent administration of 0.042-0.085 mg/kg midazolam to 18 male patients with a mean age of 68 years, mean height of 167 cm, and mean weight of 66 kg, the modified “timed up and go” (TUG) test was restored 80 minutes after administration and the blood concentration of midazolam was calculated as approximately 11.6 ng/mL. The TUG evaluates the time taken by a subject in a chair to stand up, walk, and sit back down, and is a reliable method of assessing motor ability. The studies by Pratila et al. and Fujisawa et al. were thought to delay the time to obtaining permission to return home because they used dynamic motor skills to determine the ability to return home.

This study has some limitations. One of them was a single-arm study without a control group. A comparison with a midazolam bolus or intermittent group might have revealed more benefits from continuous dosing. However, this study did not perform statistical calculations. Another one is that the blood‑level calculations rely on multiple assumptions. Therefore, we can have one of the possible results of this study, which suggests the need for further research.

## Conclusions

Midazolam was administered as an initial bolus dose of 0.05 mg/kg, followed by a continuous infusion lasting nearly 50 minutes to maintain a BIS of 70-80. If the depth of conscious sedation with midazolam is properly controlled, patients could be discharged within approximately 90 to 110 minutes of the start of midazolam administration. This means they can return home within 40-60 minutes after awakening.
